# Understanding doxorubicin associated calcium remodeling during triple-negative breast cancer treatment: an *in silico* study

**DOI:** 10.37349/etat.2021.00042

**Published:** 2021-04-30

**Authors:** Garhima Arora, Sumana Ghosh, Samrat Chatterjee

**Affiliations:** Complex Analysis Group, Translational Health Science and Technology Institute, NCR Biotech Science Cluster, Faridabad 121001, India; The University of Texas at Arlington, USA

**Keywords:** Triple-negative breast cancer, calcium signaling, mathematical model, doxorubicin, drug resistance

## Abstract

**Aim:**

Triple-negative breast cancer (TNBC) is the most malignant subtype of breast cancer with high heterogeneity, rapid progression, and paucity of treatment options. The most effective chemotherapeutic drug used to treat TNBC is doxorubicin (Doxo) which is an anthracycline antibiotic. However, Doxo treatment alters cytosolic calcium dynamics leading to drug-resistance condition. The aim of this study is to capture the alterations in the activity of various calcium channels and pumps during Doxo treatment and their consequences on cytosolic calcium dynamics that ultimately result in drug resistance.

**Methods:**

In the present study, a mathematical model is proposed to capture the complex dynamical landscape of intracellular calcium during Doxo treatment. This study provides an insight into Doxo remodeling of calcium dynamics and associated drug-resistance effect. The model was first analyzed analytically and then explored through numerical simulation using techniques like global sensitivity analysis, parameter recalibration, etc.

**Results:**

The model is used to predict the potential combination therapy for Doxo that can overcome Doxo associated drug resistance. The results show targeting the dysregulated Ca^2+^ channels and pumps might provide efficient chemotherapy in TNBC. It was also observed that the indispensability of calcium influx rate is paramount in the Doxo drug resistance. Finally, three drugs were identified from existing literature that could be used as a combination therapy along with Doxo.

**Conclusions:**

The investigation highlights the importance of integrating the calcium signaling of various calcium regulating compounds for their effective anti-tumor effects deliverance along with chemotherapeutic agents. The results from this study might provide a new direction to the experimental biologists to explore different combination therapies with Doxo to enhance its anti-tumor effect.

## Introduction

Breast cancer is the leading cause of cancer-related deaths diagnosed among women [1–3]. Triple-negative breast cancer (TNBC) is the most aggressive type of breast cancer owing to a high percentage of recurrence cases, the high incidence rate of metastasis, resulting in low survival rates. TNBC lacks the expression of commonly targeted receptors, estrogen, progesterone and human epidermal growth factor receptor 2 (HER-2) [4–7]. The proliferation rate of TNBC is higher as compared to other breast cancer subtypes [6]. TNBC is more likely to affect younger women and accounts for 10-20% of breast cancer cases diagnosed annually [2, 8]. A major clinical challenge faced in the treatment of TNBC is the lack of known specific therapeutic targets resulting in limited options to attack TNBC and hence resulting in poor prognosis. High heterogeneity in TNBC resulted in the existence of several molecular signatures, presents a significant obstacle to its successful and effective treatment [5–8]. Therefore, leaving conventional chemotherapeutics and radiation therapy to be the mainstay of TNBC treatment. Even chemotherapy with clinically recommended drugs exhibits inadequate response, high toxicity, and development of resistance [9, 10]. These challenges have encouraged much research into improving the currently available interventions and identifying an effective therapeutic strategy for TNBCs.

The most effective chemotherapeutic drug used to treat TNBC is doxorubicin (Doxo), which is an anthracycline antibiotic. The mechanism of Doxo includes the intercalation of DNA, disintegrating DNA strands, and hence inhibiting DNA and RNA synthesis. Also, Doxo inhibits the enzyme topoisomerase II, resulting in DNA damage and induction of apoptosis [11]. A recent study explained how anti-cancer drugs such as Doxo, target calcium signaling for their anti-tumor effect [12]. Moreover, Bong et al. [13] showed calcium is a central regulator of the anti-tumor properties of Doxo, thereby inducing apoptosis in a calcium-dependent manner. Abnormal calcium signaling through altered calcium channel expressions or activities results in oncogenesis and tumor development [14, 15]. Many studies done earlier showed that the anti-cancer drugs remodel calcium homeostasis, including the effect of Doxo on mitochondrial calcium in cardiomyocytes [16, 17]. Chemotherapeutic agent paclitaxel modulated endoplasmic reticulum (ER) calcium profiles in neuronal cells through inositol 1, 4, 5-trisphosphate receptor (IP_3_R) channels [18]. A study showed that the increased calcium entry through transient receptor potential canonical (TRPC)5 channel up-regulated multidrug resistance pump (P-glycoprotein) leads to resistance in MCF-7 breast cancer cells when treated with Doxo [19]. The cytosolic calcium levels increased rapidly and transiently after Doxo treatment was observed by Abdoul-Azize et al. [20] in TNBC cells. Doxo induces remodeling of calcium signaling in TNBC cells by increasing mRNA levels of calcium channels and pumps namely, calcium release-activated calcium channel protein 1 (ORAI1), TRPC1, sarco/ER Ca^2+^-ATPase 1 (SERCA1), IP_3_R2, plasma membrane Ca^2+^-ATPase 2 (PMCA2) [13].

The increased area under curve (AUC) during Doxo treatment represented the prolonged increase in cytosolic calcium levels. Also, they have revealed the dose dependent effect of Doxo. At lower Doxo dose (0.03 μM), they observed decreased cell proliferation and on higher Doxo concentration (1 μM) apoptosis was observed. These dose-dependent effects were characterized by cytosolic calcium dynamics attributes namely, peak time, peak amplitude, peak width, and AUC. However, prolonged elevation of calcium in cytosol results in oncogenesis which activates proliferation pathways and promotes tumor development [14].

The confounding effects of Doxo on intracellular calcium dynamics indicate its important role in Doxo associated drug resistance observed during TNBC treatment [21–23]. There have been many studies that have observed drug resistance by perturbing different calcium channels and pumps during Doxo treatment, however, the exact mechanism that associates Doxo-induced remodeling of cytosolic calcium dynamics with drug resistance remains to be elucidated. Our study provides an insight into Doxo remodeling of calcium dynamics and its associated drug-resistance effect. A better insight into calcium signaling and molecular changes associated with Doxo treatment can elucidate the underlying mechanism that helps to modify the cellular environment and restore the effect of Doxo treatment in TNBC. The study integrates the alterations in the activity of various calcium channels and pumps during Doxo treatment and their consequences on cytosolic calcium dynamics that ultimately result in drug resistance. We proposed a mathematical model that captures the complex dynamical landscape of intracellular calcium during Doxo treatment. The model results suggest novel combination therapies of calcium-related drugs together with Doxo for effective TNBC treatment. Finally, we identified drugs that might help in overcoming drug resistance block in Doxo treatment.

## Materials and methods

### The mathematical model

#### Background of the model

**Figure 1.**
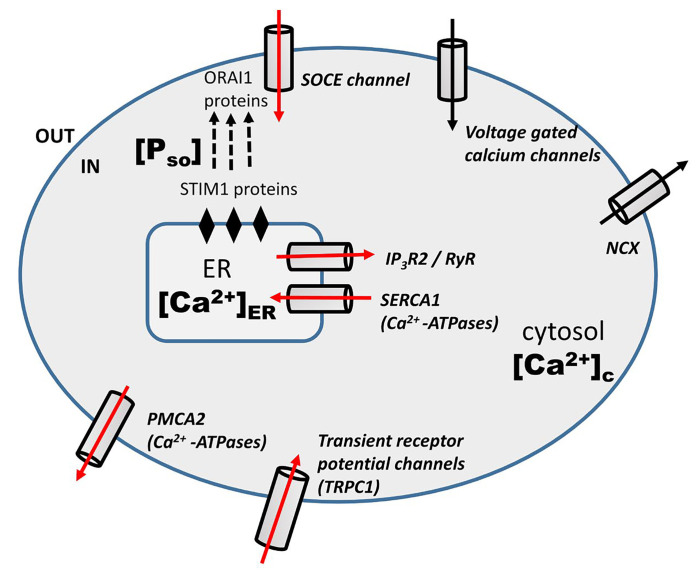
Schematic diagram: Doxo-induced modulation of calcium signaling pathways in TNBC cells [13]. The red arrow represents the increased mRNA level of calcium regulators during Doxo treatment

Doxo mediate its anti-tumor effect in a calcium-dependent manner. Doxo-induced remodeling of calcium signaling in TNBC has been observed previously [12, 13, 15, 20]. A schematic diagram (Figure 1) shows the calcium remodeling during the Doxo treatment in TNBC. Doxo treatment in TNBC increases the expression level of Ca^2+^ regulating proteins associated with calcium channels and pumps of the plasma membrane and ER namely, store-operated calcium entry (SOCE, ORAI1), TRPC1, IP_3_R2, PMCA2, and SERCA1 [13]. The increased activity of ORAI1, which is the main calcium influx channel of the plasma membrane, is connected with therapeutic resistance, invasive phenotypes, and distant metastases in many cancers [24–27]. TRPC1 is a receptor gated calcium influx channel of the plasma membrane and is included in the proliferation of various cancer cell lines [28–30]. Another plasma membrane calcium channel is PMCA2 that vacillates the calcium from the cytosol into extracellular. Its increased activity has been observed in various studies during Doxo treatment [13, 31]. However, Doxo doesn’t significantly change activities of few other calcium channels namely, voltage-gated calcium channels (VGCCs) and Na^+^/Ca^2+^ exchanger (NCX). SERCA1 and IP_3_R2 are ER influx and efflux calcium channels respectively. Their increased expressions have also been observed during Doxo treatment in studies done previously [13, 32].

#### Formulation of the model

In the last section we have clearly shown the effect of Doxo on the calcium dynamics through different channels and pumps. The involved channels and pumps are highlighted (red arrow) in the schematic diagram (Figure 1). Doxo works in a calcium-dependent manner and remodels the calcium signaling/ dynamics by increasing the calcium flux through channels and pumps, namely, SOCE (ORAI1), TRPC1, IP3R2, SERCA1, PMCA2 [13]. So in the current model, the effect of Doxo is included through parameters representing these channels and pumps. The details of parameters and terms involving these parameters are discussed below with references.

We developed a three-dimensional model using ordinary differential equations (ODEs) to capture the complex dynamical landscape of intracellular calcium during Doxo treatment. The first equation of the model describes the rate of change of cytosolic calcium level. The inward flux of Ca^2+^ from extracellular medium through VGCC is assumed to be at a constant rate *α* [33]. SOCE is one of the important plasma membrane Ca^2+^ influx channels that activate in response to [Ca^2+^]_ER_ reduction. These channels are known as SOCE. This process is intervened by STIM1 of the ER membrane and is sensitive to ER Ca^2+^ levels. STIM1 proteins bind to ORAI1, the proteins embedded in the plasma membrane that forms the pore of SOCE and triggers their opening. The inward calcium flux through the SOCE channel is defined by the term *β*[*P*_so_] [34]. As mentioned in the study done by Mirzakhalili et al. [35], the Ca^2+^ influx through TRPC1 channel is dependent on the difference between the Ca^2+^ concentration of extracellular space (*ces*) and cytosol ([Ca^2+^]_c_). Therefore, the Ca^2+^ influx through TRPC1 channel is modeled as *γ* (*c_es_* – [Ca^2+^]_c_). We assumed the Ca^2+^ concentration of extracellular to be a constant as taken by Mirzakhalili et al. [35]. The plasma membrane NCX vacillates calcium outside the cell against the gradient of sodium and the leakage from ER is supposed to follow first-order kinetics with a constant rate, *ξ* and *δ* respectively [33].

The term $\frac{p_{1}{\left[{\text{Ca}}^{2+}\right]}_{\text{c}}{\left[{\text{Ca}}^{2+}\right]}_{\text{ER}}}{q_{1}^{2}+{\left[{\text{Ca}}^{2+}\right]}_{\text{c}}^{2}}$ represents the Ca^2+^ flux into cytosol from ER through IP_3_R2 channel [33]. Here, *p*1 and *q*_1_ represents the calcium flux rate and half-saturation constant respectively for IP_3_R2 channel. SERCA1 is the calcium flux channel of ER that shuttles Ca^2+^ back into the ER and PMCA2 is the pump that vacillates calcium outside the cell. In our model, the calcium flux through these channels are represented by $\frac{p_{2}{\left[{\text{Ca}}^{2+}\right]}_{\text{c}}^{2}}{q_{2}^{2}+{\left[{\text{Ca}}^{2+}\right]}_{\text{c}}^{2}}$ and $\frac{p_{3}{\left[{\text{Ca}}^{2+}\right]}_{\text{c}}^{2}}{q_{3}^{2}+{\left[{\text{Ca}}^{2+}\right]}_{\text{c}}^{2}}$ respectively [34]. The maximum rate by which calcium vacillates into ER via SERCA1 and extracellular space through PMCA2 is represented by parameters *p*_2_ and *p*_3_ respectively. The parameters *q*_2_ and *q*_3_ describes half-saturation constants of the respective channels. The second equation describes the rate of change of calcium concentration of the ER. The calcium flux through SERCA1 and IP_3_R2 appear with the reverse sign in the second equation that represents the rate of change of ER calcium concentration. In the last equation, [*P*_so_] describes the fraction of STIM1 proteins binding to ORAI1 proteins to activate the SOCE channel for calcium influx into the cytosol from extracellular space. This fraction adjusts depending upon the changes in ER calcium levels because the diffusion of STIM1 within the ER membrane is a slow process [36]. The above phenomenon in the previous study [34] is modeled by:

$$\frac{d\left[P_{\text{SO}}\right]}{\mathrm{dt}}=\frac{P_{\text{SO}}^{\infty }\left({\left[{\text{Ca}}^{2+}\right]}_{\text{ER}}\right)-\left[P_{\text{SO}}\right]}{\tau \text{s}}\text{ where},\;P_{\text{SO}}^{\infty }\left({\left[{\text{Ca}}^{2+}\right]}_{\text{ER}}\right)=\frac{k_{\text{s}}^{4}}{k_{\text{s}}^{4}+{\left[{\text{Ca}}^{2+}\right]}_{\text{ER}}^{4}}.$$

The steady-state function $P_{\text{SO}}^{\infty }$ describes the fraction of STIM1 proteins disseminated from ER membrane (due to ER store depletion) and move towards plasma membrane to bind with ORAI1 proteins. Therefore, $P_{\text{SO}}^{\infty }$ is a decreasing function of [Ca^2+^]_ER_, and is represented by Hill function (with Hill coefficient 4), considering affinity *k*_s_ for [Ca^2+^]_ER_ [37]. Based on the above assumptions we propose the following system of differential equation,

(1) $$\begin{array}{ll} \frac{d{\left[{\text{Ca}}^{2+}\right]}_{\text{c}}}{\mathrm{dt}} & =\alpha +\beta \left[P_{\text{SO}}\right]+\gamma \left(C_{\mathrm{es}}-{\left[{\text{Ca}}^{\text{2+}}\right]}_{\text{c}}\right)+\delta {\left[{\text{Ca}}^{2+}\right]}_{\text{ER}}-\xi {\left[{\text{Ca}}^{2+}\right]}_{\text{c}}\\ & \;+\frac{p_{1}{\left[{\text{Ca}}^{2+}\right]}_{\text{c}}{\left[{\text{Ca}}^{2+}\right]}_{\text{ER}}}{q_{1}^{2}+{\left[{\text{Ca}}^{2+}\right]}_{\text{c}}^{2}}-\frac{p_{2}{\left[{\text{Ca}}^{2+}\right]}_{\text{c}}^{2}}{q_{2}^{2}+{\left[{\text{Ca}}^{2+}\right]}_{\text{c}}^{2}}-\frac{p_{3}{\left[{\text{Ca}}^{2+}\right]}_{\text{c}}^{2}}{q_{3}^{2}+{\left[{\text{Ca}}^{2+}\right]}_{\text{c}}^{2}},\\ \frac{d{\left[{\text{Ca}}^{2+}\right]}_{\text{ER}}}{\mathrm{dt}} & =\frac{p_{2}{\left[{\text{Ca}}^{2+}\right]}_{\text{c}}^{2}}{q_{2}^{2}+{\left[{\text{Ca}}^{2+}\right]}_{\text{c}}^{2}}-\frac{p_{1}{\left[{\text{Ca}}^{2+}\right]}_{\text{c}}{\left[{\text{Ca}}^{2+}\right]}_{\text{ER}}}{q_{1}^{2}+{\left[{\text{Ca}}^{2+}\right]}_{\text{c}}^{2}}-\delta {\left[{\text{Ca}}^{2+}\right]}_{\text{ER}},\\ \frac{d\left[P_{\text{SO}}\right]}{\mathrm{dt}} & =\frac{k_{\text{s}}^{4}-\left[P_{\text{SO}}\right]\left(k_{s}^{4}+{\left[{\text{Ca}}^{2+}\right]}_{\text{ER}}^{2}\right)}{\tau \text{s}\left(k_{\text{s}}^{4}+\text{b}{\left[{\text{Ca}}^{2+}\right]}_{\text{ER}}^{2}\right)} \end{array}$$

with the following initial conditions:

(2) $${\left[{\text{Ca}}^{2+}\right]}_{\text{c}}\left(0\right)={\left[{\text{Ca}}^{2+}\right]}_{\text{c}0}\geq 0;\;{\left[{\text{Ca}}^{2+}\right]}_{\text{ER}}\left(0\right)={\left[{\text{Ca}}^{2+}\right]}_{\text{ER}0}\geq 0;\left[P_{\text{so}}\right]\left(0\right)={\left[P_{\text{so}}\right]}_{0}\geq 0.$$

## Results

### Analytical results

Result 1. (i) The solution of the system (1) with respect to the initial conditions (2) exists and is unique in the interval (0, ∞) and it remains positive for all future time *t* > 0 (see Supplementary A for proof); (ii) all solutions of system (1) with positive initial conditions are uniformly bounded within a region Γ, where Γ = {([Ca^2+^]_c_, [Ca^2+^]_ER_, [*P*_so_]): 0 < [Ca^2+^]_c_ (*t*) <, $0<{\left[{\text{Ca}}^{2+}\right]}_{\text{ER}}\left(t\right)<\frac{\Lambda }{\gamma +\xi }$, $0<{\left[{\text{Ca}}^{2+}\right]}_{\text{ER}}\left(t\right)<\frac{p_{2}}{\delta }$, 0 < [ *P*_so_] (*t*) < 1}, with $\Lambda =\alpha +\beta +\gamma c_{\mathrm{es}}+P_{2}+\frac{p_{1}p_{2}}{2q_{1}\delta }$. Thus, the dynamics predicted are bounded within biological values (see Supplementary A for proof).

Result 2. The system (1) has interior equilibrium point $E^{*}\left({\left[{\text{Ca}}^{2+}\right]}_{\text{c}}^{*},{\left[{\text{Ca}}^{2+}\right]}_{\text{ER}}^{*},{\left[P_{\text{so}}\right]}^{*}\right)$ which can be obtained from the following expressions,

(3) $$\alpha +\beta {\left[P_{\text{so}}\right]}^{*}+\gamma \left(c_{\mathrm{es}}-{\left[{\text{Ca}}^{2+}\right]}_{\text{c}}^{*}\right)+\delta {\left[{\text{Ca}}^{2+}\right]}_{\text{ER}}^{*}-\xi {\left[{\text{Ca}}^{2+}\right]}_{\text{c}}^{*}+\frac{p_{1}{\left[{\text{Ca}}^{2+}\right]}_{\text{c}}^{*}{\left[{\text{Ca}}^{2+}\right]}_{\text{ER}}^{*}{}^{2}}{q_{1}^{2}+{\left[{\text{Ca}}^{2+}\right]}_{\text{c}}^{*}}-\frac{p_{2}{\left[{\text{Ca}}^{2+}\right]}_{\text{c}}^{*}{}^{2}}{q_{2}^{2}+{\left[{\text{Ca}}^{2+}\right]}_{\text{c}}^{*}{}^{2}}-\frac{p_{3}{\left[{\text{Ca}}^{2+}\right]}_{\text{c}}^{*}{}^{2}}{q_{3}^{2}+{\left[{\text{Ca}}^{2+}\right]}_{\text{c}}^{*}{}^{2}}=0$$

where, ${\left[P_{\text{so}}\right]}^{*}=\frac{k_{\text{s}}^{4}}{k_{\text{s}}^{4}+{\left[{\text{Ca}}^{2+}\right]}_{\text{ER}}^{*}{}^{4}}$, and

$${\left[{\text{Ca}}^{2+}\right]}_{\text{ER}}^{*}=\frac{p_{2}{\left[{\text{Ca}}^{2+}\right]}_{\text{c}}^{*}{}^{2}\left(q_{1}{}^{2}+{\left[{\text{Ca}}^{2+}\right]}_{\text{c}}^{*}{}^{2}\right)\left(p_{1}{\left[{\text{Ca}}^{2+}\right]}_{\text{c}}^{*}{}^{2}-\delta \left(q_{1}{}^{2}+{\left[{\text{Ca}}^{2+}\right]}_{\text{c}}^{*}{}^{2}\right)\right)}{\left(q_{2}{}^{2}+{\left[{\text{Ca}}^{2+}\right]}_{\text{c}}^{*}{}^{2}\right)}$$

The interior equilibrium point exists if we obtain the positive value of ${\left[{\text{Ca}}^{2+}\right]}_{\text{c}}^{*}$ from the expression (3) and $p_{1}>\delta \left(q_{1}^{2}+{\left[{\text{Ca}}^{2+}\right]}_{\text{c}}^{*}{}^{2}\right)$ holds. It is locally asymptotically stable if a_21_ > 0 and A_1_A_2_ > A_3_ holds simultaneously (see Supplementary A for proof).

### Choice of parameter set and validation of analytical results

A recent study done by Bong et al. [13] explored the role of Doxo through calcium signaling. They did a comparative analysis of cytosolic calcium dynamics (in MDA-MB-231 cell line) for three different conditions namely, control-treated cell line, 0.03 μM Doxo treated cell line, 1 μM Doxo treated cell line. They observed dynamics of cytosolic calcium using three criteria, which were time to reach the peak (peak time), peak amplitude, and AUC between control and Doxo treatments (0.03 μM, 1 μM). Altered calcium dynamics were observed in Doxo treated cells. The time to reach the peak and the peak amplitude of cytosolic calcium were observed between 5–20 s and 0.3–0.4 μM respectively in all three conditions. However, there was a significant difference in the AUC of control (AUC ≈ 22) and Doxo treatments (AUC ≈ 30). To study these altered calcium dynamics during Doxo treatment through our model (1), we simulated our model to replicate the above mentioned experimental results.

**Figure 2.**
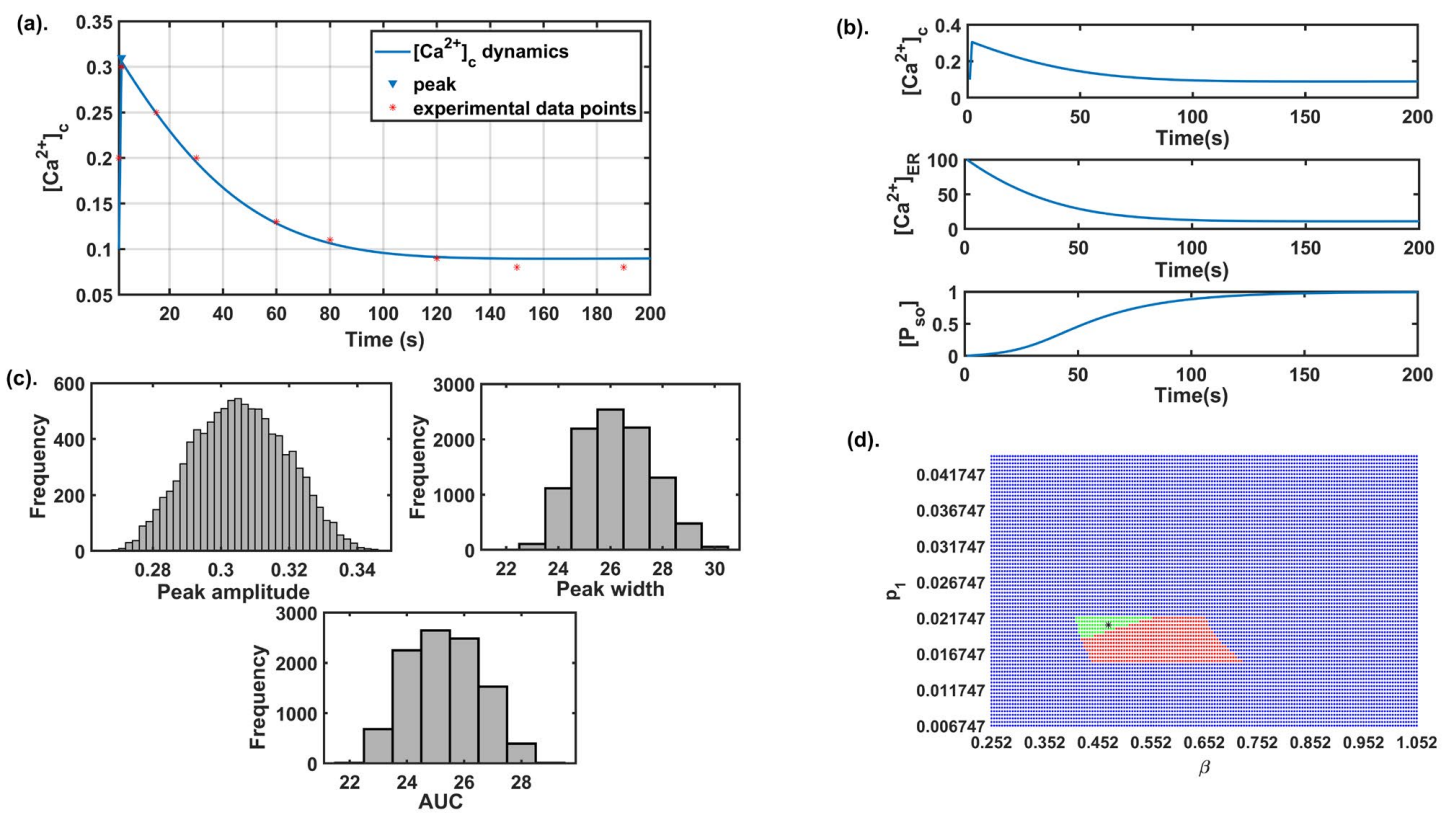
Preliminary analysis: (a) the best fit curve obtained by the least square method for estimation of parameters using experimental data [13]; (b) the dynamics of all state variables of the model for the parameter set given in Table 1; (c) the histogram plots of peak amplitude, peak width, and AUC were obtained on the parametric variation of ± 10% from the baseline; (d) the 2D parameter space between *β* and *p*1. Here, green color represents stable (focus) CRD region, red color represents stable (node) CRD region with negative real eigenvalues and blue color represents non-CRD stable region. The black star mark in the figure represents the baseline value of both parameters

We explored different literatures and obtained few parameters while the rest of the parameters namely, *β* (SOCE calcium influx rate), *γ* ([Ca^2+^] influx rate through TRPC1 channel), *δ*(leaking rate), *ξ*([Ca^2+^] export through NCX), *p*_1_ ([Ca^2+^] influx rate through IP_3_R_2_), *q*_1_ (IP_3_R_2_ channel’s half saturation constant), *p*_2_ (flux rate through SERCA1 channel) were estimated using experimental data points by Bong et al. [13]. Here we used least square method, which minimize the sum of the residuals to estimate the parameters. The base-line values of all the model parameters are given in Table 1. The model simulation results were also matched with the time to reach the peak, peak amplitude, peak width, and AUC as observed in the study [13], see Figure 2a. These cytosolic calcium dynamics are termed as control response dynamics (CRD). The dynamics of other state variables namely, [Ca^2+^]_ER_ and [*P*_SO_] are also obtained and shown in Figure 2b. A histogram study was carried out to define the ranges of model readouts (peak amplitude, peak width, and AUC) for further computational exercises in our study. We varied the base set 10% up and down which represents the neighborhood of our baseline parameter set and for which we have observed no change in the dynamical output of the system [later we explored this in detail in the robustness exercise in section (Effect of different parameters on the system dynamics)]. The parameters were varied using the Latin hypercube sampling (LHS) technique that employs Monte Carlo random sampling technique. A total of 10,000 runs were done and for each run, we calculated the peak amplitude, peak width, and AUC. A histogram plot in Figure 2c shows the range of the above three readouts establishing the range of CRD. These ranges will be used in future simulations for capturing the CRD.

**Table 1.** Parameter description and their baseline values with references

**Parameters**	**Definition**	**Value**	**Reference**
*α*	Net calcium influx rate through plasma membrane	0.04 μM/s	[38]
*β*	Maximum SOCE influx rate	0.520 μM/s	estimated
*γ*	Maximum influx of Ca^2+^ through TRPC1 channel	0.0001/s	estimated
*δ*	[Ca^2+^]_ER_ leaking rate	0.0014/s	estimated
*ξ*	Ca^2+^ export through NCX	7/s	estimated
*p*_1_	Maximal flux through IP_3_R2 channel	0.01747 μM/s	estimated
*q*_1_	IP_3_R_2_ channel’s half-saturation constant	0.0518 μM	estimated
*p*_2_	Maximal flux through SERCA1 channel	3.6926 μM/s	estimated
*q*_2_	SERCA1 channel’s half-saturation constant	0.1 μM	[39]
*p*_3_	Maximal flux through PMCA2 channel	7.34 μM/s	[34]
*q*_3_	PMCA2 channel’s half-saturation constant	1.5 μM	[34]
*c*_es_	Calcium concentration in extracellular space	1000.1812 μM	[40]
*k_s_*	STIM1 ER Ca^2+^ affinity	50 μM	[34]
*τ_s_*	SOCE timescale	30.1752 s	[34]

We also observed that for this baseline parameter set (Table 1) the existence conditions for the interior equilibrium point E* was satisfied, and the interior equilibrium point E* = (0.0903, 11.058, 0.9976) is locally asymptotically stable as the eigenvalues of the characteristic equation is (−36.5149, −0.0324 – 0.0033i, −0.0324 + 0.0033i). This follows that CRD shows stable dynamics through damped oscillations. SOCE is an important plasma membrane channel of calcium entry in the cytosol that opens in response to ER calcium depletion. Entry through this channel is mediated by STIM1 proteins that disseminate from the ER membrane and binds to ORAI1 proteins and activates SOCE. IP_3_R2 channel plays an important role in the exit of calcium from ER into cytosol. The involvement of IP_3_R2 channel in mediating SOCE has been established earlier thereby influencing cytosolic calcium dynamics [41, 42]. To observe this complex association between *β* channel’s influx rate) and *p*_1_ ([Ca^2+^] influx rate through IP_3_R2) and its role in establishing CRD. We varied these two parameters 2-fold up down from their base values and generated the 2D parameter space shown in Figure 2d. The green region represents that the system achieves CRD and stability through damped oscillations. Whereas, the system is a stable node in nature and attains CRD in the red region. We observed that on increasing *β* and setting *p*_1_ to its baseline value, the model dynamics changes from stable focus to stable node dynamics.

### Dose-dependent cytosolic calcium profiles during Doxo treatment

**Figure 3.**
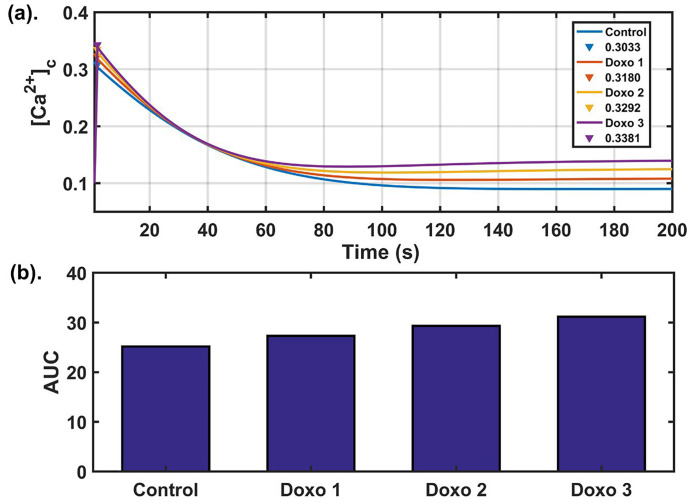
Dose dependent analysis: (a) dynamics of cytosolic calcium for four different parameter sets. The blue curve in the figure shows the cytosolic calcium dynamics obtained from baseline parameters while red, yellow, purple curves show the cytosolic calcium dynamics after increasing the parameters related to Doxo associated proteins from base values (as given in Table 1) by 25%, 50%, 75% respectively and peak values of cytosolic calcium (in μM) are also shown; (b) the bar graph shows the AUC values obtained in the four cases. Here Doxo 1, Doxo 2, Doxo 3 denotes the categories when the baseline parameter set is increased by 25%, 50%, 75% respectively

Doxo works in a concentration-dependent manner [13]. An altered calcium dynamics was observed depending on the concentration of the Doxo given in the MDA-MB-231 TNBC cell lines. They observed a significant difference in calcium dynamics of control and Doxo treated cells. Among the readouts defined in the earlier section, the most significant indicator differentiating the two cases was found to be AUC [13]. Higher AUC value in the case of Doxo represented a more sustained cytosolic calcium profile. To obtain Doxo induced calcium remodeling observed in the study done by Bong et al. [13], we varied the parameters related to Doxo associated proteins. Doxo remodels the calcium dynamics by increasing the activity/expression levels of a few calcium channels and pumps, hence increasing the calcium flux flow through these pumps and channels. To incorporate Doxo associated remodeling in our model, we also employed a similar technique to simulate such effects through our model. The parameters associated with Doxo are *β* (SOCE calcium influx rate), *γ* ([Ca^2+^] influx rate through TRPC1 channel), *p*1 ([Ca^2+^] influx rate through IP_3_R2), *p*2 (flux rate through SERCA1 channel), *p*_3_ (flux rate through PMCA2 channel), and they were simultaneously and step-wise increased by 25% (Doxo 1), 50% (Doxo 2), and 75% (Doxo 3). The calcium profile generated through elevated parameter ranges was given in Figure 3a. We found that the 75% increase in the parameters related to Doxo associated proteins reproduced the cytosolic calcium profile observed experimentally in Doxo treated cells by Bong et al. [13]. We also observed that AUC increases with every 25% step increase in the parameters related to Doxo associated proteins, see the bar graph [Figure 3b]. The AUC values for all four cases were in 90% confidence interval (CI) of the mean value (see Table 2). Moreover, the 90% CI in all four cases were non-overlapping showing that the observed trend of increase in AUC is also statistically significant (for details see Supplementary B).

**Table 2.** Readout values and 90% CI for four different parametric conditions

**Categories**	**Peak amplitude (μM)**	**Peak width (s)**	**AUC**
Control	0.3033, [0.3026, 0.3040]	26.2439, [26.2257, 26.2953]	25.1919, [25.1898, 25.2481]
Doxo 1	0.3180, [0.3173, 0.3187]	24.0503, [24.0322, 24.1164]	27.3215, [27.3201, 27.3930]
Doxo 2	0.3292, [0.3285, 0.3300]	21.3144, [21.3128, 21.3882]	29.3102, [29.3042, 29.3838]
Doxo 3	0.3381, [0.3373, 0.3390]	19.2799, [19.2700, 19.3436]	31.1513, [31.1452, 31.2368]

### Effect of different parameters on the system dynamics

In this section, we explored the effect of parameter variations on the system’s dynamics. We first varied single parameters through robustness analysis and observed their effect, and then we performed a global sensitivity analysis (GSA) to capture the effect of multi-parameter variation.

#### Robustness analysis

**Figure 4.**
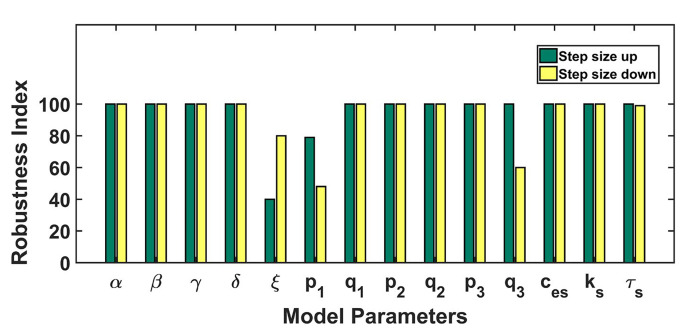
Robustness analysis: the figure shows the dependence of the model’s dynamics on parameter variations. Parameters were varied 100% up down from the baseline values. The length of bars represents the range of parameters up to which the model dynamics hold despite parameter variation. The green color bar represents the robustness result achieved by increasing the parameter value and the yellow color for the opposite case

The robustness analysis was done to assess the model’s dynamics to parameter perturbations and to establish the relevance of the obtained parameter set. To investigate whether the parametric variations disrupt the system dynamics or not, each parameter was increased and decreased one at a time by the step size of 1% of its baseline value up till 100% (see Figure 4). The bar length indicates the robustness of the parameter in conserving the model dynamics obtained from our baseline parameter set. A lower bar length specifies that a proportionately small perturbation hampered the model dynamics, while a bigger bar length specifies the robustness of the parameter in retaining the dynamical nature of the model. Our model was robustly producing the CRD against the parametric perturbations except in the case of parameters *ξ, p*1, *q*_3_ (see Table 3). Here, *ξ* represents the export of calcium into extracellular through NCX channel, p_1_ represents the calcium flux rate of IP_3_R2 channel and *q*_3_ represents half-saturation constant of PMCA2 channel.

**Table 3.** Robustness analysis table: effect of individual parameter variation on dynamics of the whole system. Here CRD and non-CRD indicate control response dynamics and non-control response dynamics respectively. By varying each parameter value individually, we observed and recorded the ranges for CRD model dynamics and non-CRD model dynamics

**Parameter**	**Range**	**Dynamics**
*α*	0 < *α* < 0.08	stable CRD
*β*	0 < *β* < 10.40	stable CRD
*γ*	0 < *γ* < 0.0002	stable CRD
*δ*	0 < *δ* < 0.0028	stable CRD
*ξ*	0 < *ξ* < 1.33	stable non-CRD
	1.33 < *ξ* < 9.8	stable CRD
	9.8 < *ξ* < 14	stable non-CRD
*p*_1_	0 < *p*_1_ < 0.009	stable non-CRD
	0.009 < *p*_1_ < 0.031	stable CRD
	0.031 < *p*_1_ < 0.035	stable non-CRD
*q*_1_	0 < *q*_1_ < 0.1036	stable CRD
*p*_2_	0 < *p*_2_ < 7.2336	stable CRD
*q*_2_	0 < *q*_2_ < 0.2	stable CRD
*p*_3_	0 < *p*_3_ < 14.68	stable CRD
*q*_3_	0 < *q*_3_ < 0.6	stable non-CRD
	0.6 < *q*_3_ < 3	stable CRD
*c_es_*	0 < *c_es_* < 2000.3624	stable CRD
*k_s_*	0 < *k_s_* < 100	stable CRD
*τ_s_*	0 < *τ_s_* < 60.3504	stable CRD

#### Global Sensitivity Analysis

The GSA identifies the efficiency of model parameters and delivers crucial details about the system performance. It encapsulates the parameters whose perturbation significantly affects/correlates the model output dynamics. Here we chose partial rank correlation coefficient (PRCC) method, which is based on sampling, for GSA. In our study, we used the LHS method as it compactly differentiates the input parameters [43]. PRCC measures the association between model inputs and outputs using correlation through the LHS method [44–46]. Each parameter was sampled over a set of values obtained by varying parameters ± 10 fold of their baseline value (Table 1). Using LHS, 10,000 sets of parameters were generated and PRCC was evaluated varying between −1 and 1. A cut off ± 0.3 was set to the sensitivity index acquired above for refining sensitive parameters [47, 48].

**Figure 5.**
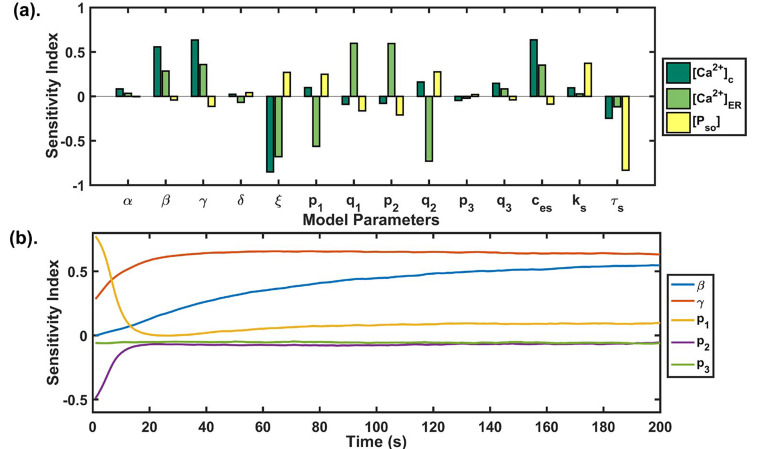
(a) GSA: the PRCC index plotted at *t* = 200 s shows the sensitivity of each model parameter against the state variables of our model. The higher the bar length, the more sensitive is the parameter to that variable. The parameter correlated negatively as well as positively with the model state variables; (b) temporal sensitivity analysis: the figure shows the temporal sensitivity analysis of parameters associated with calcium channels and pumps whose activities or expressions are significantly increased during Doxo treatment [13]

We performed sensitivity analysis for all state variables of our model (see Figure 5a). We observed that the SOCE influx rate (*β*), maximum Ca^2+^ influx through TRPC1 channel (*γ*), Ca^2+^ export through NCX (*ξ*) and calcium concentration in extracellular space (*ces*) were significantly correlated with cytosolic calcium (see Table 4). Whereas, maximum Ca^2+^ influx through TRPC1 channel (*γ*), Ca^2+^ export through NCX (*ξ*) and calcium flux rate (*p*1) and half saturation constant (*q*_1_) of IP_3_R2 channel, calcium flux rate (*p*2) and half saturation constant (*q*_2_) of SERCA1 channel were significantly correlated with [Ca^2+^]_ER_ (see Table 4).

**Table 4.** Global uncertainty analysis results: the table shows the sensitivity indexes of model parameters for all three state variables

**Parameters**	**[Ca^2+^]_c_**	**[Ca^2+^]_ER_**	**[P_SO_]**
*α*	0.0839	0.0341	−0.0048
*β*	0.5562^*^	0.2850	−0.0420
*γ*	0.6332^*^	0.3585^*^	−0.1129
*δ*	0.0230	−0.0678	0.0409
*ξ*	−0.8523^*^	−0.6793^*^	0.2704
*p*_1_	0.0986	−0.5638^*^	0.2487
*q*_1_	−0.0904	0.5954^*^	−0.1636
*p*_2_	−0.0793	0.5942^*^	−0.2100
*q*_2_	0.1606	−0.7296^*^	0.2751
*p*_3_	−0.0458	−0.0206	0.0217
*q*_3_	0.1455	0.0842	−0.0390
*c_es_*	0.6351^*^	0.3520	−0.0873
*k_s_*	0.0957	0.0277	0.3719^*^
*τ_s_*	−0.2459	−0.1178	−0.8330^*^

Each parameter was sampled for 10 fold up and down from the baseline value and obtained sensitivity indexes using PRCC algorithm. A cut-off ± 0.3 was applied over sensitivity indexes and were marked sensitive [47, 48].

* : represents the value beyond the cut-off ± 0.3

It is known that Doxo significantly increases the mRNA levels of calcium regulators associated with ORAI1, TRPC1, IP_3_R2, SERCA1, and PMCA2 [13]. Temporal sensitivity analysis was performed to study the temporal effect of the parameters (*β*, *γ*, *p*_1_, *p*2, *p*_3_) associated with these calcium channels and pumps in our model (see Figure 5b). We found that the parameters had a time-dependent sensitivity towards cytosolic calcium. We observed that two parameters *β* and p_1_ exchanged their sensitivity with time. Initially, *p*1 was sensitive but its sensitivity reduced with time, whereas the sensitivity of *β*, *γ* increased with time. The parameter *p*_2_ lost its sensitivity very fast after the first 10 s, while the parameter *p*_3_ didn’t change its sensitivity and remained insensitive throughout the time course. These results were suggesting the varying role of different calcium pumps in remodeling cytosolic calcium profile.

### Parameter recalibration

The remodeling in calcium dynamics caused by Doxo treatment in TNBC significantly increases the activity of calcium pumps and channels. This resulted in sustained cytosolic calcium levels thereby increasing the area under the curve of cytosolic calcium (see Figure 3b). This sustained calcium level in cytosol has been known to cause oncogenesis in previous reports [14, 15]. Moreover, remodeling of Ca^2+^ channels and pumps regulating proteins are connected with drug resistance in cancer [19, 49, 50]. Wang et al. [49] showed that increased activities of TRPC5 due to which multi-drug resistance pump, P-glycoprotein up-regulates in MCF-7 breast cancer cells resulted in Doxo resistance. The study showed that suppression of TRPC5 calcium channel reversed Doxo resistance in tumors formed in the MCF-7 cell line. Therefore, to mitigate the calcium-associated block in Doxo treatment, we performed parametric recalibration aiming to find parameters whose perturbations can restore CRD. We took the parameter sets which produced cytosolic calcium profiles obtained in section (Dose dependent cytosolic calcium profiles during Doxo treatment), where an increased area under the curve was observed when parameters related to Doxo associated proteins were perturbed by different step size. The range of readouts (peak amplitude, peak width, and AUC) from histogram plot (see Figure 2c) were 0.28–0.34 μM, 24–28 s and 23–27, respectively. These readout ranges are termed as CRD ranges and were further used as matching criteria for the parametric recalibration, i.e. on varying parameters, if the profile of cytosolic calcium so obtained had the above-mentioned attribute values, then it was declared that the CRD has been achieved.

**Figure 6.**
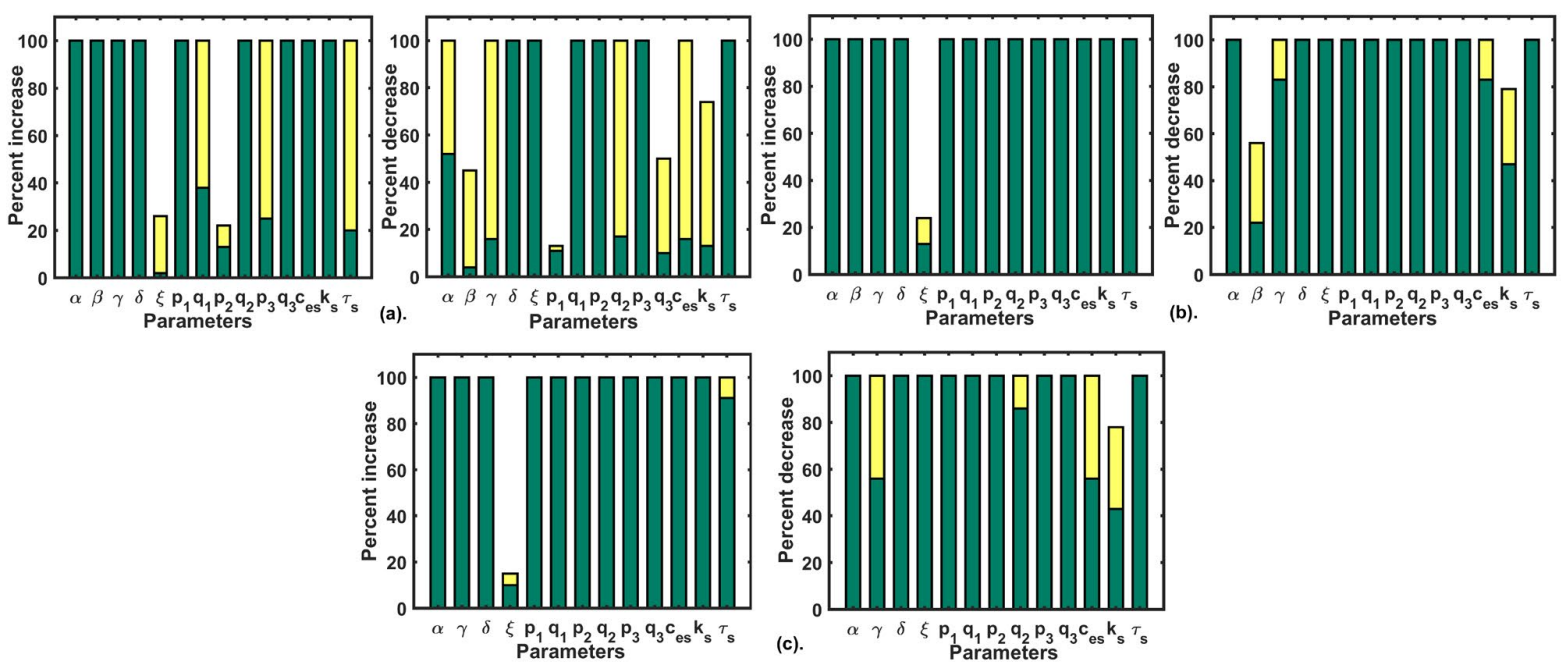
Parametric recalibration results: (a) the figure plot shows the single parameter variation results for Doxo 1; (b) the figure plot shows the single parameter variation results for Doxo 2; (c) the figure plot shows the double parameter variation results for Doxo 3 after reducing *β* (SOCE calcium influx rate) by 20%. The green bar shows the range for non-CRD and the yellow bar shows the range after CRD is obtained

We performed a single parameter variation where each parameter was perturbed 100% up-down from their respective values and we filtered out parameters that retained the Doxo profile to the control profile (CRD). This exercise was repeated for different Doxo treatments where associated parameters were increased by 25% (Doxo 1), 50% (Doxo 2), 75% (Doxo 3). Single parametric variation was able to restore the CRD only in the case of 25% and 50% increase. The results of single parameter variation for the first two cases of Doxo treatments are given in Figure 6(a–b). For parametric recalibration in the 75% case, we used two-parameter variation technique. For this rather going by brute force approach, we chose parameters related to Doxo associated proteins of our model that were, *β* (SOCE influx rate), *γ* (calcium influx rate of TRPC1), *p*1 (IP_3_R2 calcium influx rate), *p*_2_ (calcium influx rate of SERCA1), *p*_3_ (PMCA2 calcium influx rate). We picked one parameter from the above and reduced its value by 20%. After that, all other parameters were varied 100% up and down from their respective values (i.e. in the Doxo 3 parameter set) as done in the single parameter variation. Out of the five parameters related to Doxo associated proteins, the reduction of *β* (SOCE calcium influx rate) by 20% turned out to be the only parameter that can restore CRD in combination with other model parameters. Except for *β* (SOCE calcium influx rate), reduction in other parameters related to Doxo associated proteins was not sufficient to restore CRD in combination with any other model parameter. The results of double parameter variation in 75% increased case are given in Figure 6(c).

We filtered out those restoration approaches which robustly produced CRD once obtained in our parametric variation range with 90% CI of readout values (for details see Supplementary B) in the range of CRD and non-overlapping CI from their respective initial parametric conditions (Doxo 1, Doxo 2, Doxo 3). The consolidated results are shown in Table 5.

**Table 5.** Parametric recalibration table: the table describes the possible mechanisms to restore CRD under different parametric conditions. Here Doxo 1, Doxo 2, Doxo 3 denotes the categories when the baseline parameter set is increased by 25%, 50%, 75% respectively

**Category**	**Parametric variation**	**Possible mechanism of restoration**
Doxo 1	Single paramer variation	1. Decrease calcium influx rate through TRPC1 channel (*γ*)
		2. Increase SOCE timescale (*τ_s_*)
		3. Decrease net calcium influx rate through plasma membrane (*α*)
		4. Decrease extracellular calcium concentration (*c_es_*)
Doxo 2	Single parameter variation	1. Decrease TRPC1 calcium influx rate (*γ*)
		2. Decrease extracellular calcium concentration (*c_es_*)
Doxo 3	Double parameter variation	1. Decrease SOCE flux rate (*β*) by 20% & decrease TRPC1 calcium influx rate (*γ*)
		2. Decrease SOCE flux rate (*β*) by 20% & increase SOCE timescale (*τ_s_*)
		3. Decrease SOCE flux rate (*β*) by 20% & decrease SERCA1 channel’s half-saturation constant (*q*_2_)
		4. Decrease SOCE flux rate (*β*) by 20% & decrease extracellular calcium concentration (*c_es_*)

### *In silico* screening of calcium related drugs

Breast cancer cells display innate and acquired resistance to numerous anticancer drugs [51], which is the main obstacle in the effective treatment of breast cancer [52]. Therefore, enhanced medication protocols and alternative chemotherapeutic approaches are required. Studies done previously, observed the significant synergistic effects of simvastatin and Doxo together in breast cancer cells and suggested that combining simvastatin and Doxo together can be an effective treatment for breast cancer [20]. This calcium-mediated overcoming Doxo resistance encouraged us to screen known calcium-related drugs that can be administered with Doxo.

**Figure 7.**
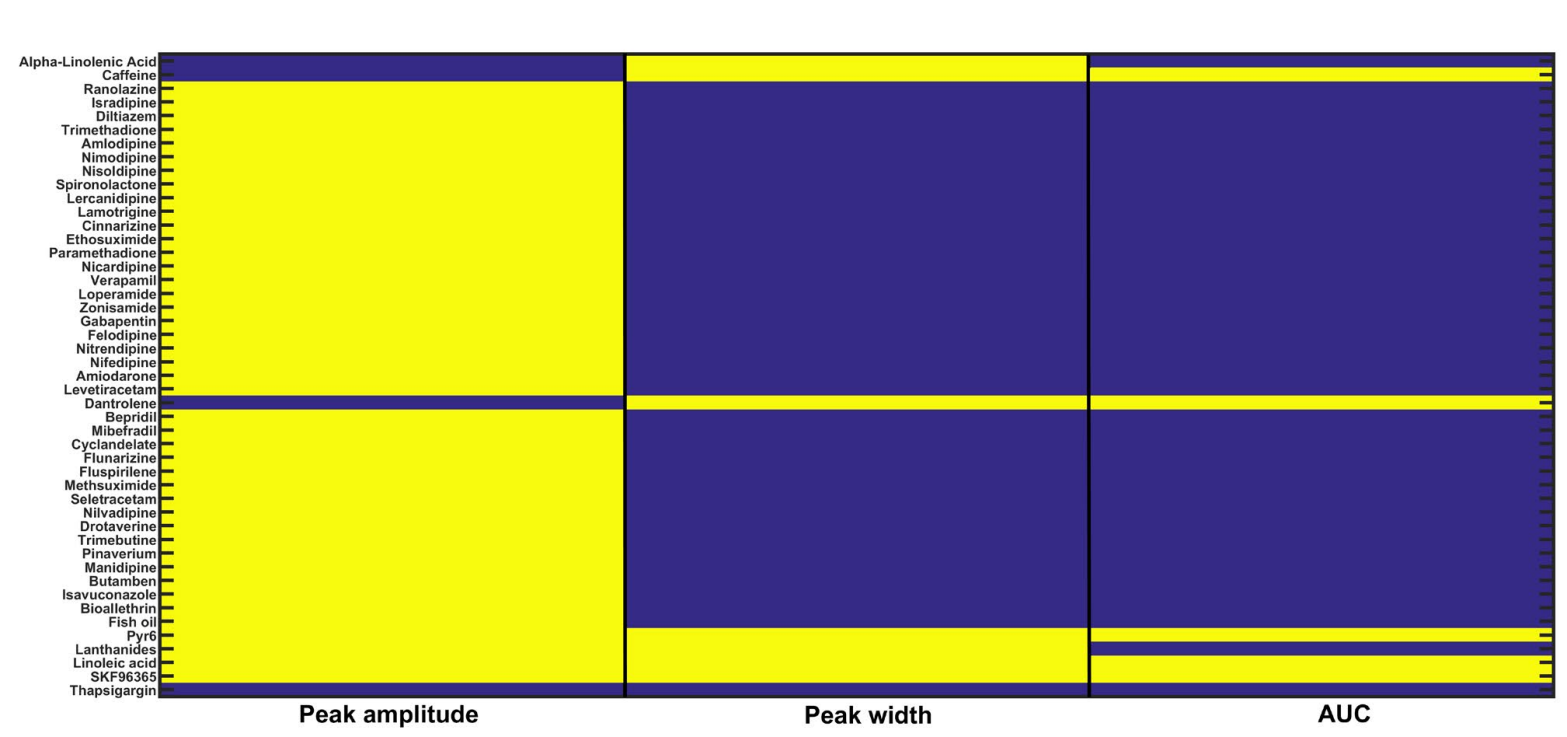
*In silico* drug screening: 47 calcium-related drugs were screened on the basis of the CRD restoration approach built-in the previous section. Three readouts namely, peak amplitude, peak width, and AUC were evaluated for each drug. The yellow color for a particular readout shows that it is in the range of CRD for the Doxo 3 + corresponding drug combination therapy and the blue color shows that it is not in the range of CRD

To execute this *in silico* experiment, we employed the CRD restoration approach (i.e. the three readout values are in the range of CRD) used in the recalibration exercise done in the previous section (Parameter recalibration). We downloaded a list of drugs with their corresponding drug targets from Drug Bank database [53] that includes approximately 13,000 drugs and their corresponding targets. We filtered out 47 drugs (inhibitors) whose targets involve Doxo associated calcium channels and pumps present in our model namely, IP3R2, SERCA1, VGCC, ORAI1, TRPC1, and NCX. We also did the literature survey and found inhibitors of calcium channels and pumps present in our study [54–56]. A complete list of drugs, drug targets, and associated parameters of our model used for further analysis is given in the Supplementary Table. We defined two cases Doxo 3 and Doxo 3 + drug in which the associated parameter with the drug is reduced by 50%. The model readouts for 47 drugs in combination with Doxo were evaluated and then examined whether CRD is restored back or not. Out of 47 drugs, Pyr6 (pyrazole compound), linoleic acid, SKF96365 showed complete restoration of CRD, i.e. corresponding to these drugs, the readout values were in the range of CRD. Dantrolene and caffeine turned out to be the only drugs with peak width and AUC values in the range of CRD. The *in silico* screening results are shown in color-coded representation (see Figure 7). The yellow color represents that the particular readout value is in the range of CRD for the corresponding drug whereas the blue color represents that the particular readout value is not in the range of CRD.

The 90% CI of readout values (see Supplementary B) was evaluated for five parametric conditions, i.e. the combination of Doxo 3 and drugs (Pyr6, linoleic acid, SKF96365, dantrolene, and caffeine). For Pyr6, linoleic acid, and SKF96365, the CI of readout values were in the range of CRD whereas, for dantrolene and caffeine, 90% CI of peak width and AUC were in the range of CRD. Moreover, in presence of the drug the CI of readouts was not overlapping with CI of readouts in the case of Doxo 3 implying a significant difference between Doxo 3 and Doxo 3 + drug (see Table 6).

**Table 6.** 90% CI for Doxo 3 and Doxo 3 + drug parametric conditions

**Cases**	**Parametric conditions**	**Peak amplitude**	**Peak width**	**AUC**
Doxo 3	75% ↑ in (*β*, *γ*, *p*_1_, *p*_2_, *p*_3_)	[0.3373, 0.3390]	[19.2700, 19.3436]	[31.1452, 31.2368]
Doxo 3 + Pyr6	Doxo 3 + 50% ↓ in *β*	[0.3271, 0.3288]	[25.1828, 25.2654]	[24.9923, 25.0537]
Doxo 3 + linoleic acid	Doxo 3 + 50% ↓ in *β* and *γ*	[0.3348, 0.3365]	[24.3140, 24.3876]	[23.2118, 23.2662]
Doxo 3 + SKF96365	Doxo 3 + 50% ↓ in *β* and *γ*	[0.3348, 0.3365]	[24.3138, 24.3892]	[23.1945, 23.2495]
Doxo 3 + dantrolene	Doxo 3 + 50% ↓ in *p*_1_	[0.2122, 0.2133]	[25.8705, 25.9780]	[26.2765, 26.3655]
Doxo 3 + caffeine	Doxo 3 + 50% ↓ in *p*_1_	[0.2122, 0.2133]	[25.8709, 25.9809]	[26.2900, 26.3800]

↓: increase in the parameter value; ↑: decrease in the parameter value

## Discussion

Calcium signaling and the regulation of intracellular calcium levels play crucial role in various life processes such as cellular growth, division, metabolism, and cell death. Whereas, alteration in calcium signaling and calcium homeostasis contributes to tumor development, proliferation, and other processes that support the progression of the tumor in various types of cancers. Doxo is the most effective chemotherapeutic drug that shows cytotoxic effects in a calcium-dependent manner towards cancer cells. Altered calcium dynamics is observed during Doxo treatment in TNBC [13, 19, 20].

### Understanding effect of Doxo on calcium dynamics through mathematical model

In the present study, we proposed a mathematical model to capture the alteration in calcium dynamics due to Doxo treatments and predict possible restoration strategies. Our model was able to replicate the experimentally observed cytosolic calcium dynamics both in control and Doxo treatments. Initially, the calcium peak was observed around 2 s and later the system showed stability through damped oscillations in the control profile that was ascertained by our analytical results. The initial peak amplitude is associated with enhanced SOCE activity and store depletion [15, 57]. The results from robustness analysis showed that our system was able to reproduce CRD against parametric perturbations except for *ξ*, *p*1, *q*_3_. The NCX exchanger together with calcium ATPase pumps plays an important role in restoring resting cytosolic calcium [15]. We defined the robust ranges for each model parameter in reproducing CRD. Moreover, through ± 10% parametric perturbation, we revealed the range of CRD in the robust parametric neighborhood.

Doxo works in a dose-dependent manner showing anti-proliferative effect at lower concentration and inducing cell apoptosis at higher doses [58, 59]. However, higher dosage of Doxo results in calcium remodeling in breast cancer cells [13, 19–21]. The Doxo associated calcium pumps and channels have taken in our model were significantly perturbed in higher dosage as compared to the lower dosage of Doxo [13]. Prolonged treatment of Doxo at higher dosage also results in overexpression of STIM/ORAI channel that has known to show anti-apoptotic effects [60]. A dose-dependent study of Doxo done by Bong et al. [13] revealed different modes of cell death based on the cytosolic calcium dynamics obtained at different Doxo doses. At higher dosage, Doxo shows its apoptotic effect in a calcium dependent manner hence this dose-dependent remodeling of calcium can affect the overall cellular fate. However, short-term and long-term effect of Doxo remains to be completely elucidated. We tried to mimic the experimentally obtained dose-dependent calcium remodeling by Doxo [13]. We evaluated AUC for every dose-dependent curve in our study. We observed that on increasing Doxo dose, i.e. on increasing the parameters related to Doxo associated proteins, AUC increased. This implies a positive correlation between Doxo doses and AUC. The dose-dependent curve obtained on increasing parameters related to Doxo associated proteins by 75%, completely replicated the curve obtained experimentally by Bong et al. [13]. The increased/prolonged cytosolic calcium during treatment is the biological relevance of increased AUC on increasing parameters related to Doxo associated proteins by 75%. The prolonged elevation of cytosolic calcium can influence the ability of cells to monitor progression through the cell cycle and lead to unchecked proliferation and tumorigenesis [61]. This motivates us to explore more on understanding the influence of parameters related to Doxo associated proteins on cytosolic calcium dynamics.

### Role of Doxo associated calcium channels and pumps

The activity of various calcium channels and pumps increases during Doxo treatment [13]. Many of these channels and pumps are associated with drug resistance [17–19]. The results of sensitivity analysis showed a high sensitivity of SOCE influx rate (*β*) and TRPC1 influx rate (*γ*) on cytosolic calcium. Moreover, these channels influx rates are among parameters related to Doxo associated proteins. The channel SOCE is an important plasma membrane calcium channel that activates in response to ER calcium depletion. SOCE is involved in various oncogenic processes [24], e.g., cancer cells invasion in glioblastoma and increase in distant metastasis in breast cancer [25]. Abdoul-Azize et al. [20] reported an increase in cytosolic calcium levels in MDA-MB-231 breast cancer cells after Doxo treatment due to increased activity of STIM1 and TRPC1. This underlines the crucial role of these channels in establishing a transient nature of cytosolic calcium during Doxo treatment. The extracellular calcium concentration also turned out to be sensitive to cytosolic calcium. This may be because, during Doxo treatment, MDA-MB-231 cells showed an enhanced sensitivity towards extracellular calcium perturbations [13]. The sensitivity analysis results portrait the calcium remodeling during Doxo treatment. Further, the temporal sensitivity analysis revealed the temporal landscape of parameters related to Doxo associated proteins for cytosolic calcium. The heterogeneity in the responses against the parameters related to Doxo associated protein perturbations at early and later time points elucidated the specific role of each calcium channel/pump on cytosolic calcium dynamics. The rise in the sensitivity of SOCE calcium influx rate (*β*) and TRPC1 calcium influx rate (*γ*) at later time points could be because they are calcium influx rates thereby, increasing cytosolic calcium levels during Doxo treatment. Interestingly, the sensitivity of ER related calcium pumps with flux rates of *p*_1_ and *p*_2_ decreased with time, and these two parameters were significantly over-expressed in Doxo treated cells [13]. The magnitude of calcium release from the ER calcium store into cytosol depends on the difference between calcium concentration of ER and cytosol [62]. Due to high influx rates from store-operated channels, the cytosolic calcium levels are elevated in Doxo treated cells. As a result, at later time points, there is a decrease in calcium concentration gradient between ER and cytosol, i.e. ER calcium levels decrease with time in correspondence to cytosolic calcium. Hence, the ER flux rates *p*_1_ and *p*_2_ become less sensitive at later time points. Each of these calcium channels and pumps modulated by Doxo holds a specific function in modulating calcium dynamics during Doxo treatment. Thus, targeting these dysregulated Ca^2+^ channels and pumps might provide efficient chemotherapy in TNBC.

### Restoration of CRD and indispensable role of SOCE (ORAI1) calcium influx rate

A major goal of the present study was to unveil possible mechanisms that can overcome the calcium-mediated Doxo drug resistance. This might lead to the identification of novel therapies. We hypothesized that restoration of calcium CRD can reduce the Doxo drug resistance. Our dose-dependent restoration approach showed diverse treatment strategies. The treatment strategies which we found relevant at lower Doxo dose were not sufficient to restore CRD at higher Doxo doses. The single parameter variation was adequate to restore back CRD for moderate cases (where parameters related to Doxo associated proteins are increased by 25% and 50%). The common restoration technique in these two cases involves a decrease in the flux through calcium channel TRPC1 (*γ*). The role of TRPC channels in calcium-related drug resistance has been reported previously where a block in these channels increased the cell sensitivity towards Doxo treatment [19]. At a higher Doxo dose, i.e. 75%, we found the indispensable role of SOCE flux rate parameter (*β*). It was only after the decrement in the value of *β* by 20%, parametric combinations were able to restore the CRD. Hence, *β* came out as the primary target in the path of overcoming calcium-mediated Doxo drug resistance. The results from the sensitivity analysis also emphasized the high calcium sensitivity against *β* perturbation and its role in cancer proliferation has been explored in studies earlier [57]. Hence, the indispensability of SOCE calcium influx rate (*β*) is paramount in the Doxo drug resistance.

### Identification of compounds as potential anti-cancer drugs in combination with Doxo

Doxo is a broad-spectrum chemotherapeutic drug used in the clinic [63]. Targeting the altered activities of Ca^2+^ channels and pumps may present promising chemotherapy for cancer treatment. The prominent research towards the assessment of various Ca^2+^ channel blockers, regulators, and inhibitors as anti-cancer drugs is emerging nowadays [14]. The *in silico* drug screening methodology employed in our study identified the drugs which together with Doxo can overcome calcium-dependent Doxo drug resistance. Calcium compounds Pyr6 (pyrazole compound), linoleic acid and SKF96365 were the only successful candidate which restored CRD. Linoleic acid is given as nutritional supplementation and for treating dietary deficiency or irregularity [64]. In a recent study, pyrazole derivatives have been considered as selective blockers of TRPC channels [65]. Pyr6 is a pyrazole compound that inhibits ORAI1 mediated calcium entry into the cytosol (represented by *β* in our model). Linoleic acid selectively inhibits the activity of the TRPC1 channel (represented by *γ* in our model) but studies have shown that it induces some STIM1-STIM1 association while inhibiting STIM1-ORAI1 binding [53, 66]. The results obtained in the drug screening exercise were in concordance with the results obtained from the recalibration exercise. The putative drug targets of the successful candidates were similar to the suggested mechanism for restoration. Linoleic acid has been screened in case of breast cancer. It has shown anti-tumor properties in breast cancer cells [67]. SKF96365 has already been known to have anti-tumor properties in a calcium-dependent manner. On treating MDA-MB-231 human breast tumor cells with SKF96365 (which blocks the store-operated [Ca^2+^] influx), the proliferation of invasive tumor cells was reduced [25]. Moreover, SKF96365 has been known to attenuate drug resistance in combination with Doxo in hepatocellular carcinoma cells [68]. This underlines the importance of this combination therapy as a putative strategy in overcoming drug-resistance.

The present study focused on the mechanistic viewpoint of reduced sensitivity of TNBC towards Doxo. The investigation highlights the importance of integrating the calcium signaling of various calcium regulating compounds for their effective anti-tumor effects deliverance along with chemotherapeutic agents. Here, we proposed few potential strategies which can be used in combination with Doxo. We further used a drug dataset to identify three drugs from existing literature that could be used individually along with Doxo as a combination therapy. Before ending this article, we would like to mention that our work lacks experimental validation, but the proposed approach delineates the complex nature of Doxo drug-resistance and can help in developing effective therapeutic strategies for TNBC.

## Abbreviations

AUC: area under curve
CI: confidence interval
CRD: control response dynamics
Doxo: doxorubicin
ER: endoplasmic reticulum
GSA: global sensitivity analysis
IP_3_R: inositol 1, 4, 5-trisphosphate receptor
LHS: Latin hypercube sampling
NCX: Na^+^/Ca^2+^ exchanger
ORAI1: calcium release-activated calcium channel protein 1
PMCA2: plasma membrane Ca^2+^-ATPase 2
PRCC: partial rank correlation coefficient
SERCA1: sarco/ER Ca^2+^-ATPase 1
SOCE: store-operated Ca^2+^ entry
STIM1: stromal interaction molecules 1
TNBC: triple-negative breast cancer
TRPC: transient receptor potential canonical
VGCCs: voltage-gated calcium channels

## Supplementary materials

The supplementary materials for this article are available at: https://www.explorationpub.com/uploads/Article/file/100242_sup_1.pdf.

## Declarations

### Copyright

© The Author(s) 2021.
