# Supplementary material for: Understanding doxorubicin associated calcium remodeling during triple-negative breast cancer treatment: an in silico study
Source: Explor Target Antitumor Ther. 2021 Apr 30;2(2):208–26. doi: 10.37349/etat.2021.00042 (PMC9400755; doi:10.37349/etat.2021.00042)
Supplement: Supplementary file 1 [file etat-02-100242-s001.pdf]

Supplementary A

Proof of Result 1. (i) To show global existence of solutions it is enough to show that the right-hand side of (1) is globally Lipschitz. The system (1) can be expressed as

$$\frac{dX}{dt} = f(X) \tag{3}$$

where $X = (x_1, x_2, x_3)^T$ and $f = (f_1, f_2, f_3)^T$. The function $f : \mathbb{R}^3_+ \to \mathbb{R}^3_+$ possesses the global Lipschitz condition if there exists a Lipschitz constant $M > 0$ such that $|f(x)-f(y)| \leq M|x-y|$ holds for any $x, y \in \mathbb{R}^3_+$.

Following [1], it can be easily proved that

$$|f_1(x) - f_1(y)| \leq M_1|x-y| \tag{4}$$
$$|f_2(x) - f_2(y)| \leq M_2|x-y| \tag{5}$$
$$|f_3(x) - f_3(y)| \leq M_3|x-y| \tag{6}$$

where $M_1 = \xi + \beta + \gamma + \delta + p_2 + p_3 + (1+K_2)p_1$, $M_2 = \delta + p_2 + (1+K_2)p_1$, $M_3 = \frac{1+k_s^4}{\tau_s}$, with the assumption that there exists real positive number K_2 such that $|x_2| \leq K_2$.

Now, to obtain global Lipschitz constant for f, we simply choose $M = \sqrt{M_1^2 + M_2^2 + M_3^2}$ and obtain

$$|f(x) - f(y)| \leq M|x-y| \tag{7}$$

Therefore, f, and hence the right-hand side of (1), is globally Lipschitz. Thus, the system possesses a unique solution. Again since f is Lipschitz, then it maps non-negative vectors to non-negative vectors, i.e., (1) gives positive invariant solution with positive initial condition (2).

(ii) From 2nd equation of system (1), we get

$$\begin{aligned}
\frac{d[Ca^{2+}]_{ER}}{dt} &= \frac{p_2[Ca^{2+}]_c^2}{q_2^2 + [Ca^{2+}]_c^2} - \frac{p_1[Ca^{2+}]_c[Ca^{2+}]_{ER}}{q_1^2 + [Ca^{2+}]_c^2} - \delta[Ca^{2+}]_{ER} \\
&\leq \max\left\{\frac{p_2[Ca^{2+}]_c^2}{q_2^2 + [Ca^{2+}]_c^2}\right\} - \delta[Ca^{2+}]_{ER} \\
&= p_2 - \delta[Ca^{2+}]_{ER}
\end{aligned}$$

So,

$$\frac{d[Ca^{2+}]_{ER}}{dt} + \delta[Ca^{2+}]_{ER} \leq p_2$$

From the theory of differential inequalities [2], we thus obtain

$$0 \leq [Ca^{2+}]_{ER}(t) \leq \frac{p_2}{\delta}\left(1 - e^{-\delta t}\right) + [Ca^{2+}]_{ER}(0)e^{-\delta t}$$

Assuming $t \to \infty$,we obtain $0 < [Ca^{2+}]_{ER}(t) < \frac{p_2}{\delta}$

Using 3rd equation of system (1), we obtain

$$\begin{aligned}
\frac{d[P_{so}]}{dt} &= \frac{k_s^4}{\tau_s\left(k_s^4+[Ca^{2+}]_{ER}^4\right)}-\frac{[P_{so}]}{\tau_s}\\
&\leq \max\left\{\frac{1}{\tau_s\left(1+\left(\frac{[Ca^{2+}]_{ER}^4}{k_s}\right)^4\right)}\right\}-\frac{[P_{so}]}{\tau_s}\\
&= \frac{1}{\tau_s}-\frac{[P_{so}]}{\tau_s}\\
\implies \frac{d[P_{so}]}{dt}+\frac{[P_{so}]}{\tau_s} &\leq \frac{1}{\tau_s}
\end{aligned} \tag{8}$$

Using the same arguments, we obtain, $0 < [P_{so}](t) < 1$ as $t \to \infty$.

From the 1st equation we obtain,

$$\begin{aligned}
\frac{d[Ca^{2+}]_c}{dt} &\leq \alpha+\beta[P_{so}]+\gamma\left(c_{es}-[Ca^{2+}]_c\right)+\delta[Ca^{2+}]_{ER}-\xi[Ca^{2+}]_c+\max\left\{\frac{p_1[Ca^{2+}]_c[Ca^{2+}]_{ER}}{q_1^2+[Ca^{2+}]_c^2}\right\}\\
&= \alpha+\beta+\gamma c_{es}+p_2+\frac{p_1p_2}{\delta}\max\left\{\frac{[Ca^{2+}]_c}{q_1^2+[Ca^{2+}]_c^2}\right\}-(\gamma+\xi)[Ca^{2+}]_c\\
&= \alpha+\beta+\gamma c_{es}+p_2+\frac{p_1p_2}{2q_1\delta}-(\gamma+\xi)[Ca^{2+}]_c \quad \left(\because\ \max\left\{\frac{[Ca^{2+}]_c}{q_1^2+[Ca^{2+}]_c^2}\right\}=\frac{1}{2q_1}\right)\\
\implies \frac{d[Ca^{2+}]_c}{dt} &+ (\gamma+\xi)[Ca^{2+}]_c \leq \Lambda, \quad \text{where,} \quad \Lambda=\alpha+\beta+\gamma c_{es}+p_2+\frac{p_1p_2}{2q_1\delta}
\end{aligned}$$

Again following [2], we can get $0 < [Ca^{2+}]_c(t) < \frac{\Lambda}{\gamma+\xi}$ as $t \to \infty$.

Hence all the solutions of the system (1) with initial condition (2) are ultimately bounded with in a region Γ, where

$$\Gamma=\left\{([Ca^{2+}]_c,[Ca^{2+}]_{ER},[P_{so}]) : 0<[Ca^{2+}]_c(t)<\frac{\Lambda}{\gamma+\xi}, 0<[Ca^{2+}]_{ER}(t)<\frac{p_2}{\delta}, 0<[P_{so}](t)<1\right\}$$

with $\Lambda=\alpha+\beta+\gamma c_{es}+p_2+\frac{p_1p_2}{2q_1\delta}$

This completes the proof.

Proof of Result 2. To study the local behaviour of the system (1) around the interior equilibrium point, we linearize the system by computing the Jacobian matrix corresponding to the interior equilibrium point $E^*\left([Ca^{2+}]_c{}^*,[Ca^{2+}]_{ER}{}^*,[P_{so}]^*\right)$.

The Jacobian matrix at the interior equilibrium point $E^*\left([Ca^{2+}]_c{}^*,[Ca^{2+}]_{ER}{}^*,[P_{so}]^*\right)$ is denoted by J_{E^*} is given by

$$J_{E^*}=\begin{pmatrix} -a_{11} & a_{12} & \beta \\ a_{21} & -a_{22} & 0 \\ 0 & -a_{32} & -\frac{1}{\tau_s} \end{pmatrix}$$

where,

$$
\begin{aligned}
a_{11} &= \gamma + \xi - \frac{p_1 q_1^2 [Ca^{2+}]_{ER}{}^* - p_1 [Ca^{2+}]_c{}^{*2} [Ca^{2+}]_{ER}{}^*}{\left(q_1^2 + [Ca^{2+}]_c{}^{*2}\right)^2} \\
&+ \frac{2 p_2 q_2^2 [Ca^{2+}]_c{}^*}{\left(q_2^2 + [Ca^{2+}]_c{}^{*2}\right)^2} + \frac{2 p_3 q_3^2 [Ca^{2+}]_c{}^*}{\left(q_3^2 + [Ca^{2+}]_c{}^{*2}\right)^2} \\
a_{12} &= \frac{p_1 [Ca^{2+}]_c{}^*}{q_1^2 + [Ca^{2+}]_c{}^{*2}}
\end{aligned}
$$

$$
\begin{aligned}
a_{21} &= \frac{2 p_2 q_2^2 [Ca^{2+}]_c{}^*}{\left(q_2^2 + [Ca^{2+}]_c{}^{*2}\right)^2} - \frac{p_1 q_1^2 [Ca^{2+}]_{ER}{}^* - p_1 [Ca^{2+}]_c{}^{*2} [Ca^{2+}]_{ER}{}^*}{\left(q_1^2 + [Ca^{2+}]_c{}^{*2}\right)^2} \\
a_{22} &= \frac{p_1 [Ca^{2+}]_c{}^*}{q_1^2 + [Ca^{2+}]_c{}^{*2}} + \delta \\
a_{32} &= \frac{4 k_s^4 [Ca^{2+}]_{ER}{}^{*3}}{\tau_s \left(k_s^4 + [Ca^{2+}]_{ER}{}^{*4}\right)^2}
\end{aligned}
$$

Now the characteristic equation of the Jacobian matrix is as follows,

$$
\lambda^3 + A_1 \lambda^2 + A_2 \lambda + A_3 = 0 \tag{9}
$$

where,

$$
\begin{aligned}
A_1 &= \gamma + \xi + \delta + \frac{1}{\tau_s} - \frac{p_1 q_1^2 [Ca^{2+}]_{ER}{}^* - p_1 [Ca^{2+}]_c{}^{*2} [Ca^{2+}]_{ER}{}^*}{\left(q_1^2 + [Ca^{2+}]_c{}^{*2}\right)^2} \\
&+ \frac{2 p_2 q_2^2 [Ca^{2+}]_c{}^*}{\left(q_2^2 + [Ca^{2+}]_c{}^{*2}\right)^2} + \frac{2 p_3 q_3^2 [Ca^{2+}]_c{}^*}{\left(q_3^2 + [Ca^{2+}]_c{}^{*2}\right)^2} + \frac{p_1 [Ca^{2+}]_c{}^*}{q_1^2 + [Ca^{2+}]_c{}^{*2}} \\
A_2 &= \left(\gamma + \xi - \frac{p_1 q_1^2 [Ca^{2+}]_{ER}{}^* - p_1 [Ca^{2+}]_c{}^{*2} [Ca^{2+}]_{ER}{}^*}{\left(q_1^2 + [Ca^{2+}]_c{}^{*2}\right)^2} + \frac{2 p_2 q_2^2 [Ca^{2+}]_c{}^*}{\left(q_2^2 + [Ca^{2+}]_c{}^{*2}\right)^2} + \frac{2 p_3 q_3^2 [Ca^{2+}]_c{}^*}{\left(q_3^2 + [Ca^{2+}]_c{}^{*2}\right)^2}\right) \\
&\left(\delta + \frac{1}{\tau_s}\right) + \frac{\delta}{\tau_s} + \left(\gamma + \xi + \frac{1}{\tau_s} + \frac{2 p_3 q_3^2 [Ca^{2+}]_c{}^*}{\left(q_3^2 + [Ca^{2+}]_c{}^{*2}\right)^2}\right) \frac{p_1 [Ca^{2+}]_c{}^*}{q_1^2 + [Ca^{2+}]_c{}^{*2}} \\
A_3 &= \frac{\delta}{\tau_s} \left(\gamma + \xi - \frac{p_1 q_1^2 [Ca^{2+}]_{ER}{}^* - p_1 [Ca^{2+}]_c{}^{*2} [Ca^{2+}]_{ER}{}^*}{\left(q_1^2 + [Ca^{2+}]_c{}^{*2}\right)^2} + \frac{2 p_2 q_2^2 [Ca^{2+}]_c{}^*}{\left(q_2^2 + [Ca^{2+}]_c{}^{*2}\right)^2} + \frac{2 p_3 q_3^2 [Ca^{2+}]_c{}^*}{\left(q_3^2 + [Ca^{2+}]_c{}^{*2}\right)^2}\right) \\
&+ \left(\gamma + \xi + \frac{2 p_3 q_3^2 [Ca^{2+}]_c{}^*}{\left(q_3^2 + [Ca^{2+}]_c{}^{*2}\right)^2}\right) \frac{p_1 [Ca^{2+}]_c{}^*}{\tau_s \left(q_1^2 + [Ca^{2+}]_c{}^{*2}\right)} \\
&+ \left(\frac{2 p_2 q_2^2 [Ca^{2+}]_c{}^*}{\left(q_2^2 + [Ca^{2+}]_c{}^{*2}\right)^2} - \frac{p_1 q_1^2 [Ca^{2+}]_{ER}{}^* - p_1 [Ca^{2+}]_c{}^{*2} [Ca^{2+}]_{ER}{}^*}{\left(q_1^2 + [Ca^{2+}]_c{}^{*2}\right)^2}\right) \frac{4 k_s^4 [Ca^{2+}]_{ER}{}^{*3}}{\tau_s \left(k_s^4 + [Ca^{2+}]_{ER}{}^{*4}\right)^2}
\end{aligned}
$$

Now if $a_{21} > 0$ i.e.,

$$\frac{2p_2q_2^2[Ca^{2+}]_c{}^*}{\left(q_2^2+[Ca^{2+}]_c{}^{*2}\right)^2} > \frac{p_1q_1^2[Ca^{2+}]_{ER}{}^* - p_1[Ca^{2+}]_c{}^{*2}[Ca^{2+}]_{ER}{}^*}{\left(q_1^2+[Ca^{2+}]_c{}^{*2}\right)^2}$$

then $A_1, A_2, A_3 > 0$. Hence following Routh-Hurwitz criterion, the local asymptotically stability condition of interior equilibrium $E^*\left([Ca^{2+}]_c{}^*, [Ca^{2+}]_{ER}{}^*, [P_{so}]^*\right)$ is $A_1A_2 > A_3$. Hence proved.

Supplementary B

Statistical significance analysis of model readouts

To evaluate the statistical significance of model readouts (obtained in section 4.2) for the four different parametric conditions (Control, Doxo 1, Doxo 2, Doxo 3), we varied doxorubicin associated parameters 10% up-down. A total of 1000 sample parameter sets were generated using Latin hypercube sampling (LHS) in 10% neighbourhood of the test parameter set. We calculated the readout values namely, peak amplitude, peak width, and AUC for 1000 sample sets generated above, and evaluated the 90% confidence interval (CI) for the mean of these readouts. The readout values for different parametric conditions (Control, Doxo 1, Doxo 2, Doxo 3) given in Table (2) lied in the 90% confidence interval of the mean value. Moreover, the CI of AUC in all four conditions was not overlapping implying that the AUC is significantly different in all four conditions [3].

A similar methodology was applied to other places to study the statistical significance through CI.

References

1. Knipl DH. Fundamental properties of differential equations with dynamically defined delayed feedback. Electron J Qual Theory Differ Equ. 2013;17:1-18.
2. Birkhoff G, Rota GC. Ordinary differential equations. 4th ed. New York: Wiley; 1978.
3. Cumming G. Inference by eye: reading the overlap of independent confidence intervals. Stat Med. 2009;28:205-20.

Table

Serial number	Drug ID	Drug
D1	DB00132	Alpha-Linolenic Acid
D2	DB00201	Caffeine
D3	DB00243	Ranolazine
D4	DB00270	Isradipine
D5	DB00343	Diltiazem
D6	DB00347	Trimethadione
D7	DB00381	Amlodipine
D8	DB00393	Nimodipine
D9	DB00401	Nisoldipine
D10	DB00421	Spironolactone
D11	DB00528	Lercanidipine
D12	DB00555	Lamotrigine
D13	DB00568	Cinnarizine
D14	DB00593	Ethosuximide
D15	DB00617	Paramethadione
D16	DB00622	Nicardipine
D17	DB00661	Verapamil
D18	DB00836	Loperamide
D19	DB00909	Zonisamide
D20	DB00996	Gabapentin
D21	DB01023	Felodipine
D22	DB01054	Nitrendipine
D23	DB01115	Nifedipine
D24	DB01118	Amiodarone
D25	DB01202	Levetiracetam
D26	DB01219	Dantrolene
D27	DB01244	Bepridil
D28	DB01388	Mibefradil
D29	DB04838	Cyclandelate
D30	DB04841	Flunarizine
D31	DB04842	Fluspirilene
D32	DB05246	Methsuximide
D33	DB05885	Seletracetam
D34	DB06712	Nilvadipine
D35	DB06751	Drotaverine
D36	DB09089	Trimebutine
D37	DB09090	Pinaverium
D38	DB09238	Manidipine
D39	DB11148	Butamben
D40	DB11633	Isavuconazole
D41	DB13746	Bioallethrin
D42	DB13961	Fish oil
D43		Pyr6
D44		Lanthanides
D45		Linoleic acid
D46		SKF96365
D47		Thapsigargin

Pump/Channel (Targets)	Associated parameters in our model
NCX	ξ
IP_3R	p_1
VGCC	α
VGCC	α
VGCC	α
VGCC	α
VGCC	α
VGCC	α
VGCC	α
VGCC	α
VGCC	α
VGCC	α
VGCC	α
VGCC	α
VGCC	α
VGCC	α
VGCC	α
VGCC	α
VGCC	α
VGCC	α
VGCC	α
VGCC	α
VGCC	α
VGCC	α
VGCC	α
RyR1	p_1
VGCC	α
VGCC	α
VGCC	α
VGCC	α
VGCC	α
VGCC	α
VGCC	α
VGCC	α
VGCC	α
VGCC	α
VGCC	α
VGCC	α
VGCC	α
VGCC	α
VGCCC	α
VGCCC	α
ORAI1	β
VGCC, ORAI1, TRPC1	α, β, γ
ORAI1, TRPC1	β, γ
ORAI1,TRPC	β, γ
SERCA	p_2

Mode of action
inhibitor
inhibitor
inhibitor
inhibitor
inhibitor/ blocker
inhibitor
inhibitor
inhibitor
inhibitor
inhibitor
inhibitor
inhibitor
inhibitor
inhibitor
inhibitor
inhibitor
inhibitor
inhibitor
inhibitor
inhibitor
inhibitor
inhibitor
inhibitor
inhibitor
inhibitor
inhibitor
inhibitor
inhibitor
inhibitor
inhibitor
inhibitor
inhibitor
inhibitor/ blocker
inhibitor
inhibitor
inhibitor
inhibitor
inhibitor/ blocker
inhibitor
inhibitor
inhibitor/ blocker
inhibitor
inhibitor
inhibitor
inhibitor
inhibitor
inhibitor
